# First isolation of two colistin-resistant emerging pathogens, *Brevundimonas diminuta *and *Ochrobactrum anthropi*, in a woman with cystic fibrosis: a case report

**DOI:** 10.1186/1752-1947-2-373

**Published:** 2008-12-05

**Authors:** Magalie Menuet, Fadi Bittar, Nathalie Stremler, Jean-Christophe Dubus, Jacques Sarles, Didier Raoult, Jean-Marc Rolain

**Affiliations:** 1 URMITE UMR 6236, CNRS-IRD, Faculté de Médecine et de Pharmacie, Bd Jean Moulin, 13385 Marseille cedex 05, France; 2 Département des Maladies respiratoires, centre de Ressources et de compétences pour la Mucoviscidose Enfants (CRCM), Hôpital Timone, Marseille, France

## Abstract

**Introduction:**

Cystic fibrosis afflicted lungs support the growth of many bacteria rarely implicated in other cases of human infections.

**Case presentation:**

We report the isolation and identification, by 16S rRNA amplification and sequencing, of two emerging pathogens resistant to colistin, *Brevundimonas diminuta *and *Ochrobactrum anthropi*, in a 17-year-old woman with cystic fibrosis and pneumonia. The patient eventually responded well to a 2-week regime of imipenem and tobramycin.

**Conclusion:**

Our results clearly re-emphasize the emergence of new colistin-resistant pathogens in patients with cystic fibrosis.

## Introduction

Cystic fibrosis (CF) is one of the most common autosomal-recessive hereditary diseases in Europeans and is characterized by disorders of the respiratory tract and pancreas, and exacerbations of pulmonary infections. A limited number of organisms are responsible for these infections, with *Staphylococcus aureus *and *Pseudomonas aeruginosa *being of primary importance. Recent studies, using molecular approaches, have identified uncommon bacteria and/or novel pathogens in patients with CF [1] including strains resistant to colistin such as *Stenotrophomonas maltophilia, Achromobacter xylosoxidans, Burkholderia cepacia *and *Inquilinus limosus *[2]. While the frequency of infection with these species is believed to be relatively low and their significance unclear, they present a real challenge to diagnostic laboratories, as they are difficult to identify and often misidentified as belonging to the *Burkholderia cepacia *complex [1,3]. We report the isolation and identification, by 16S rRNA sequencing, of two emerging pathogens resistant to colistin, *Brevundimonas diminuta *and *Ochrobactrum anthropi *in a 17-year-old patient with cystic fibrosis and pneumonia. The study was approved by the local ethics committee (IFR48).

## Case presentation

A 17-year-old woman with cystic fibrosis, and with diabetes and persistent colonization of the respiratory tract with *Staphylococcus aureus *since childhood was admitted in October 2006 to our specialized centre for a respiratory infection with dark sputum, asthenia, fever (38.5°C) and a loss of weight of 4.5 kg. On examination, the patient had shortness of breath and diffuse crepitations in both lungs. The oxygen saturation on air was 92% and her chest X-ray showed a diffuse bronchitis syndrome with bronchial distension in the right lung apex and left lung base. There was no pleural effusion. Relevant laboratory findings included a white blood cell (WBC) count of 18,380/mm^3 ^with 82.8% polymorphonuclear cells (PMNs), a platelet count of 618,000/mm^3^, C-reactive protein (CRP) of 57 mg/litre, fibrinogen of 5.17 g/litre and whole blood glucose of 9 mmol/litre. An admission sputum sample was plated onto Columbia colistin-nalidixic acid (CNA) agar, chocolate Poly ViteX agar, MacConKey agar (bioMérieux, Marcy l'Etoile, France), CEPACIA agar, and SABOURAUD agar (AES laboratory, Combourg, France). Direct Gram staining of the sputa showed numerous PMNs (>25 cells/field), Gram-positive cocci, and infrequent epithelial cells (<10 cells/field). Apart from 10^7 ^CFU/ml methicillin-susceptible *S. aureus*, two different Gram-negative rods (oxidase and catalase positive) were isolated from CEPACIA agar at 10^3 ^CFU/ml after 3 days of incubation. Using API 20NE (bioMérieux, Marcy l'Etoile, France), two isolates initially identified as *Weeksella virosa */*Empedobacter brevis *(Code 0010014, 84.5% probability) and *Ochrobactrum anthropi *(code 1641344, 98.9% probability) were definitively identified as *B. diminuta *(100% homology with *B. diminuta *strain DSM 1635, GenBank accession number X87274) and *O. anthropi *(100% homology with *O. anthropi *strain W24, GenBank accession number EF198140), respectively, after amplification and sequencing of the 16S rRNA gene as previously described [4]. Although there is neither clear consensus nor guidelines for antibiotic susceptibility testing (AST) of these two bacteria, AST was performed using VITEK 2 Auto system (bioMérieux, Marcy l'Etoile, France) and disc diffusion methods. The *B. diminuta *was resistant to amoxicillin, amoxicillin/clavulanic acid, ceftazidime, ciprofloxacin, trimethoprim/sulphamethoxazole and colistin but remained susceptible to ceftriaxone, ticarcillin, ticarcillin/clavulanic acid, imipenem, amikacin, tobramycin, gentamicin, isepamicin, rifampicin, and piperacillin/tazobactam. The *O. anthropi *was resistant to amoxicillin, amoxicillin/clavulanic acid, ticarcillin, ticarcillin/clavulanic acid, ceftazidime, ceftriaxone, piperacillin/tazobactam and colistin but remained susceptible to ciprofloxacin, imipenem, amikacin, tobramycin, gentamicin, isepamicin, trimethoprim/sulphamethoxazole and rifampicin. The patient was initially treated with ceftazidime (2 g 4 times/day) and nebulized tobramycin (300 mg/day) for 2 weeks. The treatment was switched to intravenous imipenem (4 g/day) and tobramycin (320 mg/day) for 2 weeks with dramatic improvement. Two weeks later, the patient was clinically well and sputum culture yielded a mixed oral population. *B. diminuta *and *O. anthropi *were not cultured again in 5 sputa investigated during 7 months of follow-up.

## Discussion

**Figure 1:**
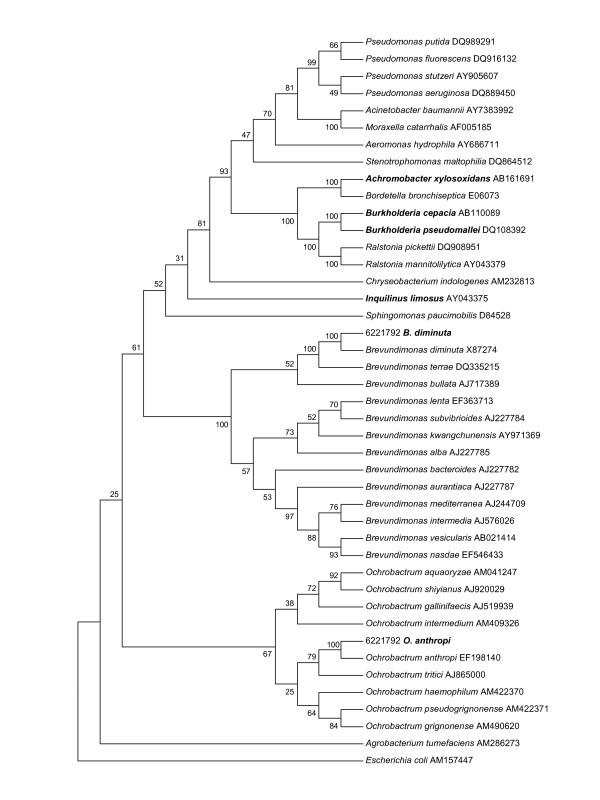
Phylogenetic tree based on 16S rRNA sequences. The information presented includes bacterial species or phylotype and GenBank accession number. Footnote: Bacteria that are given in bold have been described as colistin-resistant in patients with cystic fibrosis.

*B. diminuta *is a non-lactose-fermenting environmental Gram-negative bacillus previously assigned to the genus *Pseudomonas *(Figure 1) that has been occasionally implicated in clinical situations in immunocompetent and immunocompromised hosts including bacteraemia, urinary infection and emphysema [5,6]. In a study by Kiska *et al*. [3], *B. diminuta *was isolated in a patient with cystic fibrosis, after being misidentified as *B. cepacia*, but the identification was not performed using molecular methods and the patient's clinical condition was not reported [3]. *O. anthropi *is a Gram-negative non-fermenting bacillus widely distributed in the environment that has rarely been reported as a human pathogen. It has been implicated in several clinical situations in immunocompetent and immunocompromised hosts including osteochondritis, necrotizing fasciitis, endophthalmitis, cellulitis, sepsis, chest wall abscess, osteomyelitis, endocarditis and pelvic abscess [7-9]. *O. anthropi *is characterized by a broad spectrum of antibiotic resistance and is believed to be naturally susceptible to colistin [10] whereas there are currently no available data for AST of *B. diminuta*. It should be noted that our patient received a course of colistin to treat a *A. xylosoxidans *colonization 10 months before the onset of this pneumonia. This may have contributed to the selection of these two colistin-resistant bacteria in our patient. We believe that these two colistin-resistant pathogens were the main cause of her acute pneumonia. Although *S. aureus *may also partially participate in the pathogenic process, *O. anthropi *and *B. diminuta *were isolated during this pneumonia. Moreover, the patient did not improve initially with an effective antibiotherapy against *S. aureus *(ceftazidime) and improved using an effective antibiotic treatment against the two colistin/ceftazidime-resistant strains suggesting a role of one or both colistin-resistant strains as an agent of lower respiratory tract infection in this patient.

## Conclusion

Our results clearly re-emphasize the emergence of new colistin-resistant pathogens in patients with cystic fibrosis as recently reported for *Inquilinus limosus *[2]. The increased clinical use of nebulized colistin in patients with cystic fibrosis may select specific colistin-resistant bacteria. Furthermore, the use of *Burkholderia cepacia *complex selective agar associated with molecular approaches may allow the identification of emerging colistin-resistant pathogens in patients with cystic fibrosis.

## Abbreviations

AST: antibiotic susceptibility testing; CF: cystic fibrosis; CNA: Columbia colistin-nalidixic acid; CRP: C-reactive protein; PMNs: polymorphonuclear cells; WBC: white blood cell.

## Consent

Written informed consent was obtained from the patient for publication of this case report and any accompanying images. A copy of the written consent is available for review by the Editor-in-Chief of this journal.

## Competing interests

The authors declare that they have no competing interests.

## Authors' contributions

MM and FB collected the data and drafted the manuscript. NS, JCD and JS took care of the patient during hospitalization. DR and JMR participated in the design and critical revision of the study and helped to draft the manuscript. All authors read and approved the final manuscript.
